# Long-term outcomes and prognostic predictors in patients with fibrosing mediastinitis associated pulmonary hypertension: a multicenter cohort study

**DOI:** 10.1186/s13023-025-04066-8

**Published:** 2025-11-12

**Authors:** Hanwen Zhang, Beilan Yang, Bingyang Liu, Qin Luo, Yinjiang Tang, Ting Wang, Gaxue Jiang, Qunying Xi, Yinli Wang, Hongyun Ruan, Jinglong Nan, Yicheng Yang, Qixian Zeng, Wenjie Yan, Yanru Liang, Zhihui Zhao, Tao Yang, Yu Chen, Yan Wu, Xincao Tao, Xiaoming Zhou, Zhihong Liu, Lingyan You, Changming Xiong

**Affiliations:** 1 Respiratory and Pulmonary Vascular Center, State Key Laboratory of Cardiovascular Disease, Fuwai Hospital, National Center for Cardiovascular Diseases, Chinese Academy of Medical Sciences and Peking Union Medical College, North Lishi Road, Xicheng District, No. 167, Beijing, 100037 China; 2 Yunnan Provincial Cardiovascular Disease Clinical Medical Center, Affiliated Cardiovascular Hospital of Kunming Medical University, Kunming, Yunnan Province 650102 China; 3 Inner Mongolia People’s Hospital, Inner Mongolia Autonomous Region, Hohhot, 010010 China; 4 Cardiac Center, The First Hospital of Lanzhou University, Lanzhou, Gansu 730000 China; 5 Pulmonary Vascular Diseases General Ward, Fuwai Hospital Chinese Academy of Medical Sciences, 12 Langshan Rd, Shenzhen, Guangdong Province 518057 China; 6 The Third People’s Hospital of Chengdu, Chengdu, Sichuan Province 610014 China; 7 Xuzhou Central Hospital, Xuzhou, Jiangsu Province 221009 China

**Keywords:** Fibrosing mediastinitis, Pulmonary hypertension, Predictors, Prognosis

## Abstract

**Background:**

Fibrosing mediastinitis (FM) is a rare condition that may be complicated by pulmonary hypertension (PH). This multicenter study aimed to investigate the clinical features, long-term survival outcomes, and prognostic factors in patients with fibrosing mediastinitis associated pulmonary hypertension (FM-PH).

**Methods:**

A total of 85 FM-PH patients were enrolled across seven centers in China between January 2007 and July 2024. Patients were classified into two groups based on the occurence of clinical worsening (CW): FM-PH with CW and FM-PH without CW. Clinical worsening was defined as a composite of all-cause mortality, rehospitalization for heart failure, or deterioration in World Health Organization functional class (WHO-FC) compared with baseline.

**Results:**

Of the 85 FM-PH patients, 37 were classified as FM-PH without CW and 48 as FM-PH with CW. The FM-PH with CW patients had significantly higher levels of systemic inflammation, elevated N-terminal pro-brain natriuretic peptide (NT-proBNP), higher mean pulmonary artery pressure (mPAP), and worse right heart function compared to the FM-PH without CW patients. Multivariate Cox regression analysis showed that high-sensitivity C-reactive protein (hs-CRP), mPAP, and peripheral edema were independently associated with clinical worsening in FM-PH patients. Over a median follow-up of 27 months [IQR 11–55], 48 patients experienced clinical worsening events, including five deaths. The 1-, 3-, and 5-year overall survival rates were 94.5%, 86.3%, and 84.6%, respectively. However, the 5-year clinical worsening-free rate was only 26.6%.

**Conclusion:**

Although the 1-, 3-, and 5-year overall survival rates were relatively favorable, the 5-year clinical worsening-free rate was not satisfying. Elevated hs-CRP, mPAP and the presence of peripheral edema were independently associated with worse clinical outcomes.

**Supplementary Information:**

The online version contains supplementary material available at 10.1186/s13023-025-04066-8.

## Introduction

Fibrosing mediastinitis (FM) is a rare, slowly progressive and potentially fatal disorder characterized by excessive proliferation of fibrotic tissue and chronic inflammation within the mediastinum [1, 2]. This pathological process can lead to compression or obstruction of mediastinal structures, including the pulmonary arteries, pulmonary veins, superior vena cava (SVC), and esophagus [3]. The etiology and underlying molecular mechanisms of FM remain incompletely understood. In North America, *Histoplasma capsulatum* infection is the most common cause, typically affecting young adults without a gender predilection [4]. In contrast, tuberculosis infection is the primary cause of FM in China, where it predominantly affects the elderly populations [5]. The clinical manifestations of FM are typically nonspecific and highly variable, largely depending on which mediastinal structures are involved and the extent of disease progression.

Fibrosing mediastinitis associated pulmonary hypertension (FM-PH), is a frequent and serious complication of FM and is categorized as Group 5 pulmonary hypertension [6]. It results from progressive external compression of the pulmonary vessels, leading to increased pulmonary arterial pressure and vascular resistance. Over time, this can cause right heart failure and ultimately death. The prognosis is poor, with reported 1-, 3-, and 5-year survival rates of 88%, 73%, and 56%, respectively [7]. Owing to the rarity of the disease and the nonspecific nature of its symptoms, FM-PH is often misdiagnosed, and clinical awareness remains low. Early recognition and appropriate management are therefore critical to improving patient outcomes.

Therefore, this multicenter cohort study aimed to enhance the understanding of FM-PH by evaluating its clinical characteristics, long-term survival outcomes, and prognostic factors in a large patient population.

## Methods

### Study design

This retrospective study included both incident and prevalent cases of FM-PH diagnosed between January 2007 and July 2024 at seven centers across 23 provinces in China. The diagnosis was based on computed tomography pulmonary angiography (CTPA) and relevant pulmonary hemodynamic assessments. Inclusion criteria were as follows: (1) evidence of an infiltrative fibrotic process in the mediastinum causing compression of major structures on CTPA, supported by thorough clinical evaluation; and (2) right heart catheterization (RHC) showing a mean pulmonary artery pressure (mPAP) greater than 20 mmHg. Exclusion criteria included: (1) incomplete clinical data; and (2) pulmonary artery involvement due to alternative etiologies such as mediastinal malignancy, pulmonary vasculitis, sarcoidosis, IgG4-related disease, or prior radiation therapy.

The study was conducted in accordance with the Declaration of Helsinki and received approval from the ethics committees of all participating institutions (approval number: 2025–2662). Written informed consent was obtained from all participants.

### Data sources

Baseline data were retrospectively collected at the time of initial FM-PH diagnosis, which was defined as the date of the first RHC meeting the diagnostic criteria for FM-PH. Clinical information was obtained from electronic medical records by two independent reviewers and included demographics, clinical presentation, comorbidities, WHO functional class (WHO-FC), laboratory findings, blood gas analysis, pulmonary function tests, use of pulmonary arterial hypertension (PAH)-specific medications, history of balloon pulmonary angioplasty (BPA) or percutaneous pulmonary vascular stenting, and parameters from echocardiography, CT, and invasive hemodynamic assessments. Standard transthoracic echocardiography was performed following American Society of Echocardiography (ASE) guidelines [8]. The echocardiographic diagnosis of right heart enlargement was made based on more than 95% confidence interval of normal dimensions of right atrium (RA) or right ventricle (RV).Patients were categorized based on clinical worsening for comparative analyses. Management decisions were made by multidisciplinary teams consisting of cardiologists, pulmonologists, and radiologists, incorporating both medical and interventional therapies.

### Clinical assessment and follow-up

Follow-up data were obtained through outpatient visits, rehospitalization records, and telephone interviews. The primary endpoint was clinical worsening, defined as a composite of all-cause mortality, rehospitalization for heart failure, or deterioration in WHO-FC compared with baseline.

### Statistical analyses

Continuous variables were presented as mean ± standard deviation for normally distributed data or as median with interquartile range (IQR) for non-normally distributed data. Categorical variables were expressed as frequencies and percentages. Group comparisons were performed using the independent t-test or Mann–Whitney U test for continuous variables, and the chi-square test or Fisher’s exact test for categorical variables.

Survival duration was measured from the time of diagnosis to either the occurrence of a primary endpoint or the date of the last available follow-up. To evaluate the association between continuous high-sensitivity C-reactive protein (hs-CRP) and mPAP values with clinical worsening, restricted cubic spline curves were applied. According to the cut-off values identified from these curves, hs-CRP and mPAP were categorized into high and low groups. Kaplan–Meier survival curves were constructed, and group differences were assessed using the log-rank test. Variables showing a univariate association with survival (*P* < 0.05) were further analyzed using multivariable Cox proportional hazards models with backward-stepwise elimination. All statistical analyses were performed using R software (version 4.4.3; R Foundation for Statistical Computing), with statistical significance defined as a two-sided P-value < 0.05.

## Results

### Patients selection

**Fig. 1:**
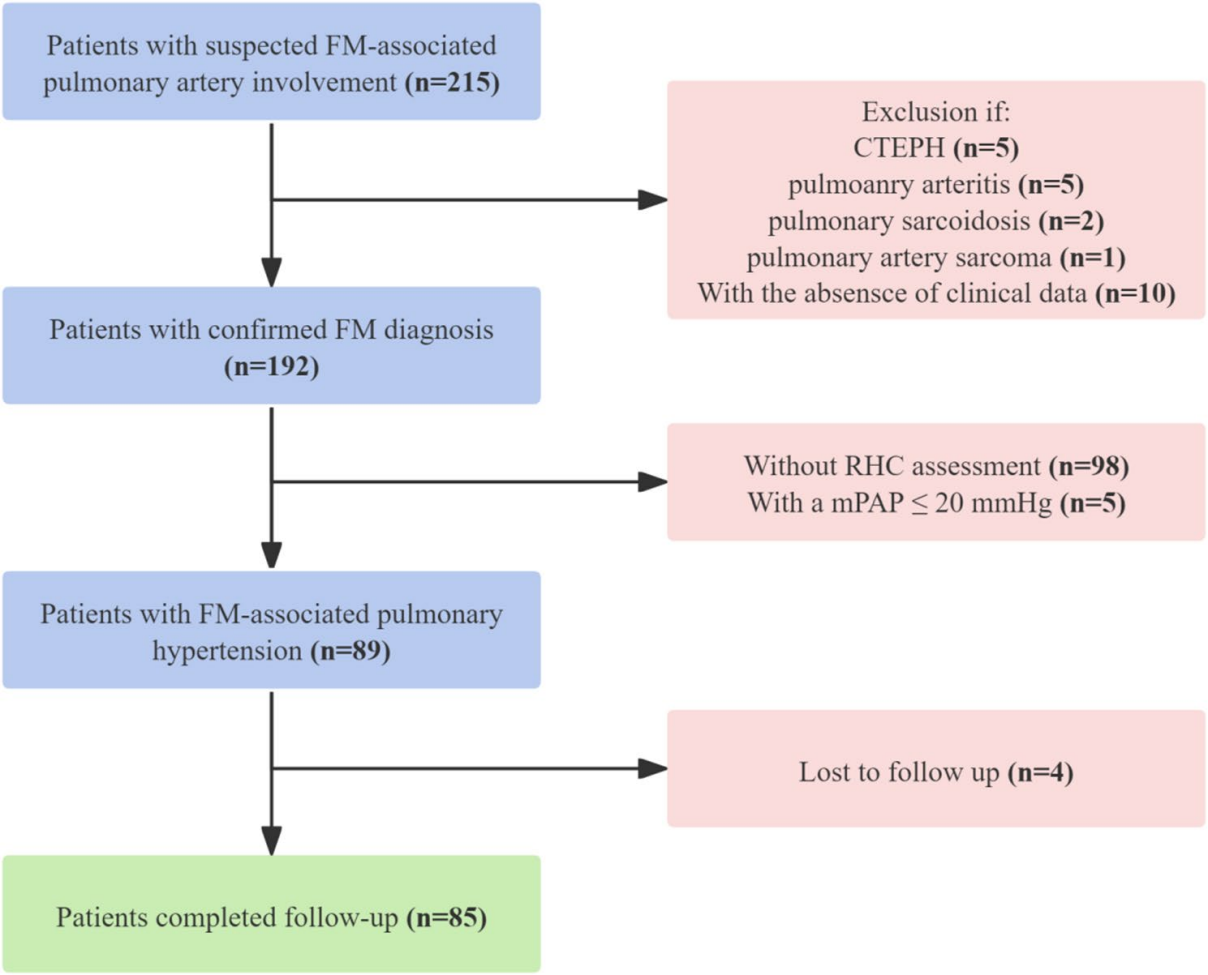
Flowchart of patient selection for the study. CTEPH, chronic thromboembolic pulmonary hypertension; FM, fibrosing mediastinitis; mPAP, mean pulmonary artery pressure; RHC, right heart catheterization

The patient selection process is illustrated in Fig. 1. Of the 215 patients initially suspected of having FM, 23 were excluded—10 due to missing clinical data and 13 due to alternative causes of pulmonary artery stenosis (including five with chronic thromboembolic pulmonary hypertension, five with pulmonary arteritis, two with pulmonary sarcoidosis, and one with pulmonary artery sarcoma). An additional 103 patients were excluded: 98 due to the absence of invasive hemodynamic measurements and five due to a baseline mPAP of ≤ 20 mmHg. Four patients lost to follow-up were also excluded. Ultimately, 85 patients who met the diagnostic criteria for FM-PH and had complete follow-up data were included in the final analysis.

### Clinical characteristics

Most patients in this study were female (64.7%), with a median age of 67.0 years [IQR: 60.0–71.0]. Of the 85 participants, 37 were classified into the FM-PH-without-CW group, and 48 into the FM-PH-with-CW group. The clinical characteristics of both groups are summarized in Table 1. In the FM-PH-with-CW group, 15 patients (31.9%) were in WHO-FC I–II, while 32 patients (68.1%) were in class III-IV. Dyspnea was the most common symptom, reported by 88.2% of patients, followed by cough (43.5%) and palpitations (30.6%). A prior history of tuberculosis was documented in 27 patients (31.8%), 13 of whom had completed anti-tuberculosis treatment before their FM-PH diagnosis. No patients had active tuberculosis at the time of enrollment. Additionally, silicosis was reported in five patients (5.9%). The underlying etiology remained unidentified in the remaining cases. Additionally, compared with the FM-PH-without-CW group, the FM-PH-with-CW group showed significantly higher levels of several inflammatory and cardiac biomarkers: white blood cell count (6.5 × 10^9^/L [IQR 5.7–8.1] vs. 5.8 × 10^9^/L [IQR 4.8–6.8], *P* = 0.011), neutrophil count (4.5 × 10^9^/L [IQR 3.6–5.6] vs. 3.7 × 10^9^/L [IQR 2.7–4.3], *P* = 0.007), monocyte count (0.4 × 10^9^/L [IQR 0.3–0.5] vs. 0.3 × 10^9^/L [IQR 0.3–0.4], *P* = 0.034), CRP (4.8 mg/L [IQR 2.5–8.0] vs. 2.1 mg/L [IQR 1.5–3.4], *P* = 0.003), hs-CRP (3.3 mg/L [IQR 1.2–5.5] vs. 1.2 mg/L [IQR 0.6–3.7], *P* = 0.008), and NT-proBNP levels (337.0 pg/mL [IQR 123.9–778.2] vs. 130.0 pg/mL [IQR 88.4–434.0], *P* = 0.019).

**Table 1:** Demographic and clinical characteristics in 85 patients with FM-PH

Characteristics	Total, *n* = 85	Non-clinical worsen, *n* = 37	Clinical worsen, *n* = 48	*p*.value
**Clinical characteristics**				
Age, years	67.0 (60.0–71.0)	67.0 (63.0–70.0)	66.0 (58.5–71.0)	0.496
Male, n(%)	30 (35.3%)	15 (39.5%)	15 (31.9%)	0.619
BMI	22.2 (20.4–25.0)	22.9 (21.5–24.8)	21.2 (20.0–25.0)	0.186
Smoking, n (%)	22 (25.9%)	12 (31.6%)	10 (21.3%)	0.407
WHO-FC, n(%)				0.142
I-II	51(60.0%)	19(50.0%)	15 (31.9%)	
III-IV	34 (40.0%)	19 (50.0%)	32(68.1%)	
***Symptoms***				
Dyspnea, n(%)	75 (88.2%)	35 (92.1%)	40 (85.1%)	0.501
Cough, n(%)	37 (43.5%)	18 (47.4%)	19 (40.4%)	0.673
Palpitation, n(%)	26 (30.6%)	12 (31.6%)	14 (29.8%)	1.000
Expectoration, n(%)	25 (29.4%)	13 (34.2%)	12 (25.5%)	0.526
Peripheral edema, n(%)	21 (24.7%)	6 (15.8%)	15 (31.9%)	0.144
Fatigue, n(%)	20 (23.5%)	7 (18.4%)	13 (27.7%)	0.459
Hemoptysis, n(%)	17 (20.0%)	7 (18.4%)	10 (21.3%)	0.957
Chest pain, n(%)	15 (17.6%)	10 (26.3%)	5 (10.6%)	0.110
***Comorbidities***				
Tuberculosis, n(%)				0.615
Confirmed	25 (29.4%)	11 (28.9%)	14 (29.8%)	
Suspected	2 (2.4%)	0 (0.0%)	2 (4.3%)	
Silicosis, n(%)	5 (5.9%)	3 (7.9%)	2 (4.3%)	0.652
***Laboratory data***				
NT-proBNP, pg/ml	244.0 (103.8-599.5)	130.0 (88.4–434.0)	337.0 (123.9-778.2)	0.019*
White blood cell, 10^9^/L	6.2 (5.1–7.5)	5.8 (4.8–6.8)	6.5 (5.7–8.1)	0.011*
Neutrophil, 10^9^/L	4.0 (3.3-5.0)	3.7 (2.7–4.3)	4.5 (3.6–5.6)	0.007*
Lymphocyte, 10^9^/L	1.6 (1.3-2.0)	1.5 (1.2–1.8)	1.6 (1.3–2.2)	0.429
Monocyte, 10^9^/L	0.4 (0.3–0.5)	0.3 (0.3–0.4)	0.4 (0.3–0.5)	0.034*
C-reactive protein, mg/L	3.0 (1.8–6.4)	2.1 (1.5–3.4)	4.8 (2.5-8.0)	0.003*
hs-CRP, mg/L	2.5 (0.9-5.0)	1.2 (0.6–3.7)	3.3 (1.2–5.5)	0.008*
PaCO_2_, mmHg	36.1 (33.8–39.9)	35.2 (34.0-40.5)	37.0 (33.8–39.6)	0.948
PaO_2_, mmHg	73.8 ± 15.2	76.8 ± 16.0	71.3 ± 14.1	0.080
SaO_2_, %	94.6 (91.1–95.8)	95.0 (92.3–96.0)	94.2 (90.8–95.2)	0.126
***Echocardiography***				
Right heart enlargement, n(%)^a^	51 (60.0%)	17 (44.7%)	34 (72.3%)	0.018*
RA, mm	41.0 (37.0–43.0)	38.0 (36.0–42.0)	42.0 (38.0–45.0)	0.021*
RVED, mm	27.0 (24.0–30.0)	27.0 (24.2–30.0)	26.0 (23.0–29.0)	0.278
LVED, mm	41.0 (39.0–45.0)	42.0 (40.2–45.8)	41.0 (38.0-44.5)	0.068
RV/LV	0.6 (0.5–0.7)	0.6 (0.5–0.7)	0.6 (0.5–0.7)	0.968
D_MPA_, mm	27.0 (24.0–32.0)	27.5 (24.0–32.0)	27.0 (23.5–30.0)	0.285
sPAP, mmHg	65.0 (45.0–84.0)	59.0 (41.0–74.0)	75.0 (51.0–92.0)	0.013*
LVEF,%	65.0 (61.0–69.0)	64.5 (61.2–69.0)	65.0 (60.0–68.0)	0.375
V_TR_,m/s	3.9 (3.2–4.5)	3.7 (3.1–4.2)	4.2 (3.3–4.6)	0.051
Pericardial effusion, %	8 (9.4%)	4 (10.5%)	4 (8.5%)	1.000
***Pulmonary function***, ***n* = 69**				
TLC, % pred	76.0 (65.0-82.5)	75.5 (65.0-81.8)	76.0 (67.0–83.0)	0.609
FVC, % pred	84.0 (72.5–99.0)	82.0 (74.0–92.0)	85.0 (71.5-100.8)	0.459
FEV1, % pred	65.0 (49.0–83.0)	64.0 (48.0-85.5)	65.0 (53.0–80.0)	0.805
FEV1/FVC	65.1 ± 13.1	64.5 ± 15.2	65.5 ± 11.4	0.770
DLCO, % pred	58.5 (48.8–75.5)	58.0 (46.5–77.5)	60.0 (51.0–73.0)	0.849
***Pulmoanry hemodynamics***				
mRAP, mmHg	5.0 (3.2-7.0)	6.0 (4.8-8.0)	5.0 (3.0–7.0)	0.134
mPAP, mmHg	34.0 (29.0–44.0)	33.0 (27.2–42.0)	39.0 (30.5–46.0)	0.047*
PAWP, mmHg	9.6 (7.0–12.0)	10.0 (8.0-12.8)	9.0 (6.5–12.0)	0.110
Cardiac index, L/min/m^2^	2.8 (2.3–3.5)	2.8 (2.5–3.8)	2.6 (2.3–3.4)	0.255
PVR, wood units	6.9 (4.1–10.3)	5.2 (3.6–8.7)	8.2 (5.3–10.8)	0.017*
SvO_2_, %	72.1 (66.2–75.9)	72.9 (66.6–79.0)	71.5 (66.0-74.2)	0.185
SpaO_2_, %	70.4 (64.4–75.8)	71.6 (66.3–78.8)	68.7 (64.1–72.1)	0.062
SaO_2_, %	94.2 (91.1–95.5)	94.5 (91.7–95.7)	93.9 (90.8–95.2)	0.179
PAC, mL/mmHg	0.1 (0.1–0.2)	0.1 (0.1–0.2)	0.1 (0.1–0.2)	0.111
***Treatments***				
BPA combined with PAH-specific therapy, n(%)	20 (23.5%)	11 (28.9%)	9 (19.1%)	0.423
BPA, n(%)	41 (48.2%)	20 (52.6%)	21 (44.7%)	0.609
PAH-specific therapy, n(%)	41 (48.2%)	20 (52.6%)	21 (44.7%)	0.609
Monotherapy	34 (40.0%)	16 (42.1%)	18 (38.3%)	0.894
PDE5i	17 (20.0%)	4 (10.5%)	13 (27.7%)	0.091
sGC	16 (18.8%)	11 (28.9%)	5 (10.6%)	0.062
ERA	13 (15.3%)	7 (18.4%)	6 (12.8%)	0.677
Prostanoids	1 (1.2%)	1 (2.6%)	0 (0.0%)	0.447
Combination therapy	7 (8.2%)	4 (10.5%)	3 (6.4%)	0.695

Data are presented as mean ± standard deviation, median (25th–75th percentile) or number (percentage)

*with a P value less than 0.05

BMI, body mass index; BPA, balloon pulmonary angioplasty; DLCO, diffusion capacity of carbon monoxide; D_MPA_, diameter of main pulmonary artery; ERA, endothelin receptor antagonist; FVC, forced vital capacity; FEV1, forced expiratory value in 1 s; FM-PH, fibrosing mediastinitis associated pulmonary hypertension; hs-CRP, high-sensitivity C-reactive protein; LVED, left ventricular end-diastolic diameter; LVEF, left ventricular ejection fraction; mRAP, mean right atrial pressure; mPAP, mean pulmonary artery pressure; NT-proBNP, N-terminal pro-brain natriuretic peptide; PAC, pulmonary arterial compliance; PaCO_2_, partial pressure of carbon dioxide; PaO_2_, partial pressure of oxygen; PAWP, pulmonary artery wedge pressure; PDE-5i, phosphodiesterase type-5 inhibitors; PH, pulmonary hypertension; PVR, pulmonary vascular resistance; RA, right atrial minor dimension; RVED, right ventricular end-diastolic diameter; RV/LV, right ventricular end-diastolic diameter/left ventricular end-diastolic diameter; SaO_2_, arterial oxygen saturation; sGC, soluable guanylate cyclase; sPAP, systolic pulmonary artery pressure; SpaO_2,_ pulmonary arterial oxygen saturation; SvO_2_, mixed venous oxygen saturation; TLC, total lung capacity; V_TR_, velocity of tricuspid regurgitation; % pred, percent of predicted value; WHO-FC, World Health Organization functional class

a Right heart enlargement was defined qualitatively as RA or RV dilation observed on echocardiography

All patients underwent standardized echocardiographic assessment. Systolic pulmonary arterial pressure (sPAP) was significantly higher in the FM-PH-with-CW group compared with the FM-PH-without-CW group (75.0 mmHg vs. 59.0 mmHg, *P* = 0.013). Right heart enlargement was more frequently observed in the FM-PH-with-CW group (72.3% vs. 44.7%, *P* = 0.018), and the right atrial (RA) minor dimension was markedly larger in the CW cohort (42.0 mm [IQR 38.0–45.0] vs. 38.0 mm [IQR 36.0–42.0], *P* = 0.021). Left ventricular systolic function was preserved across all patients, with a median ejection fraction of 65.0% [IQR 61.0–69.0]. Pericardial effusion was present in 9.4% of patients, suggesting potential right ventricular decompensation. However, there were no significant differences between the two groups in terms of right ventricular end-diastolic diameter, left ventricular end-diastolic diameter, the RV/LV ratio, main pulmonary artery diameter, left ventricular ejection fraction, tricuspid regurgitation velocity, or incidence of pericardial effusion.

Pulmonary function test results were available for 69 patients. Obstructive ventilatory defects were the most common (*n* = 28), followed by mixed (*n* = 40) and restrictive (*n* = 1) patterns. However, no significant differences were observed in TLC%pred, FVC%pred, FEV1%pred, FEV1/FVC ratio, or diffusing capacity for carbon monoxide percent predicted (DLCO%pred) between patients when stratified by clinical worsening status.

Hemodynamic assessments showed significantly higher mPAP) in the FM-PH-with-CW group compared with the FM-PH-without-CW group (39.0 mmHg [IQR 30.5–46.0] vs. 33.0 mmHg [IQR 27.2–42.0], *P* = 0.047), along with increased pulmonary vascular resistance (PVR) (8.2 Wood units [IQR 5.3–10.8] vs. 5.2 Wood units [IQR 3.6–8.7], *P* = 0.017). According to standardized classification criteria, 80 patients were diagnosed with isolated pre-capillary pulmonary hypertension, while five patients had combined pre- and post-capillary pulmonary hypertension (CpcPH). No cases of isolated post-capillary pulmonary hypertension were identified.

In terms of treatment, 41 patients (48.2%) received medications approved for PAH, including 34 on PAH-specific monotherapy and 7 on combination therapy. BPA was performed in 41 patients (48.2%), and 3 patients underwent percutaneous vascular stent implantation. Phosphodiesterase type-5 inhibitors (PDE-5i) were the most frequently used drug in monotherapy (20.0%), followed by soluable guanylate cyclase (sGC) (18.8%) and endothelin receptor antagonist (ERA) (15.3%). A combined pharmacological and interventional approach was employed in 20 patients (23.5%).

### CTPA imaging characteristics

All 85 patients underwent chest CT and CTPA at baseline. Radiologic findings are summarized in Table 2. Bilateral lesions were observed in 91.2% of cases, while left-sided and right-sided involvement were rare, accounting for only 6.2% and 2.5% of cases, respectively. Compression of the main pulmonary artery (MPA) was identified in 8.2% of patients, with a median MPA diameter of 31.5 mm (IQR 29.0–35.0); no significant differences were observed between the two groups. Lobar vascular compression was most common in the right middle lobe (71.8%), followed by the right upper (65.9%), right lower (65.9%), left lower (62.4%), and left upper (58.8%) lobes. Pulmonary vein compression was identified in 12 patients (14.1%), with no significant difference between the FM-PH-with-CW and FM-PH-without-CW groups (*P* = 0.877). Bronchial compression was another prominent feature, with 84.7% of patients showing involvement of the lobar bronchi and 12.9% exhibiting compression of the main bronchi. Among other mediastinal structures, SVC compression was observed in only 2.4% of patients, while esophageal involvement was rare, occurring in just one patient (1.2%).

**Table 2:** Radiological characteristics on computed tomography in 85 patients with FM-PH

Characteristics	Total, *n* = 85	Non-clinical worsen, *n* = 37	Clinical worsen, *n* = 48	*p*.value
Anatomic distribution of FM within the mediastinum, n(%)				0.826
Left	5 (6.2%)	3 (8.3%)	2 (4.5%)	
Right	2 (2.5%)	1 (2.8%)	1 (2.3%)	
Bilateral	73 (91.2%)	32 (88.9%)	41 (93.2%)	
Pulmonary arteries compression				
MPA compression, n(%)	7 (8.2%)	3 (7.9%)	4 (8.5%)	1.000
MPA diameter, mm	31.5 (29.0–35.0)	30.0 (28.0–35.0)	32.0 (30.0–36.0)	0.261
RPA diameter, mm	22.0 (20.0–25.0)	21.0 (18.0–25.0)	22.0 (20.0–25.0)	0.337
LPA diameter, mm	20.0 (17.0-22.8)	20.0 (17.0–22.0)	20.0 (18.0–23.0)	0.482
Lobar arteries compression				
LUL compression, n(%)	50 (58.8%)	23 (60.5%)	27 (57.4%)	0.948
LLL compression, n(%)	53 (62.4%)	20 (52.6%)	33 (70.2%)	0.150
RUL compression, n(%)	56 (65.9%)	23 (60.5%)	33 (70.2%)	0.480
RML compression, n(%)	61 (71.8%)	27 (71.1%)	34 (72.3%)	1.000
RLL compression, n(%)	56 (65.9%)	22 (57.9%)	34 (72.3%)	0.243
PV compression, n(%)	12 (14.1%)	6 (16.2%)	6 (12.5%)	0.877
Bronchial compression				
Main bronchi compression, n(%)	11 (12.9%)	7 (18.4%)	4 (8.5%)	0.207
Lobar bronchi compression, n(%)	72 (84.7%)	31 (81.6%)	41 (87.2%)	0.742
SVC compression, n(%)	2 (2.4%)	1 (2.6%)	1 (2.1%)	1.000
Esophagus compression, n(%)	1 (1.2%)	0 (0.0%)	1 (2.1%)	1.000
MLN enlargement, n(%)	79 (92.9%)	36 (94.7%)	43 (91.5%)	0.687
MLN position, n(%)				0.404
Left	7 (8.2%)	5 (13.2%)	2 (4.3%)	
Right	1 (1.2%)	0 (0.0%)	1 (2.1%)	
Bilateral	72 (84.7%)	31 (81.6%)	41 (87.2%)	
MLN fusion, n(%)	76 (89.4%)	34 (89.5%)	42 (89.4%)	1.000
MLN calcification, n(%)	52 (61.2%)	27 (71.1%)	25 (53.2%)	0.145
BHLN calcification, n(%)	55 (64.7%)	28 (73.7%)	27 (57.4%)	0.184
Pulmonary fibrosis, n(%)	29 (34.1%)	11 (28.9%)	18 (38.3%)	0.500
Pleural thickening, n(%)	30 (35.3%)	12 (31.6%)	18 (38.3%)	0.677
Lung micro nodule, n(%)	59 (69.4%)	25 (65.8%)	34 (72.3%)	0.678
Atelectasis, n(%)	40 (47.1%)	18 (47.4%)	22 (46.8%)	1.000
Ground glass nodule, n(%)	50 (58.8%)	18 (47.4%)	32 (68.1%)	0.088
Pleural effusion, n(%)	13 (15.3%)	5 (13.2%)	8 (17.0%)	0.850

Data are presented as mean ± standard deviation, median (25th–75th percentile) or number (percentage)

BHLN, bilateral hilar lymph nodes; FM-PH, fibrosing mediastinitis associated pulmonary hypertension; LLL, left lower lobe; LPA, left pulmonary artery; LUL, left upper lobe; MLN, mediastinal lymph nodes; MPA, main pulmonary artery; PV, pulmonary vein; RLL, right lower lobe; RML, right middle lobe; RPA, right pulmonary artery; RUL, right upper lobe; SVC, superior vena cava

Mediastinal lymphadenopathy was common, occurring in 92.9% of patients, with bilateral lymph node enlargement observed in 84.7%. Most of the enlarged lymph nodes were fused (89.4%), and calcification was frequently present (61.2%). Bilateral hilar lymph node calcification was noted in 64.7% of cases. Parenchymal findings included lung micronodules (69.4%), ground-glass opacities (58.8%), atelectasis (47.1%), pleural thickening (35.3%), pulmonary fibrosis (34.1%), and pleural effusion (15.3%). However, no significant differences in imaging features were found between the two groups.

### Risk factors for clinical worsening

The results of the univariate Cox regression analysis are presented in Table 3. Variables significantly associated with a higher risk of clinical worsening included NT-proBNP, hs-CRP, white blood cell count, monocyte count, arterial oxygen saturation (SaO_2_), mPAP, PVR, RV/LV, and peripheral edema (all *P* < 0.05). In the backward-stepwise multivariate Cox regression analysis, three independent predictors were identified: increased hs-CRP (hazard ratio [HR] = 1.08, 95% confidence interval [CI] 1.02–1.15, *P* = 0.010), increased mPAP (HR = 1.03, 95% CI 1.01–1.06, *P* = 0.017), and the presence of peripheral edema (HR = 1.93, 95% CI 1.01–3.71, *P* = 0.040), all of which were independently associated with clinical worsening. Subgroup analyses stratified by age, sex, BMI, smoking status, and tuberculosis history (Supplementary Fig. 1) showed consistent associations for hs-CRP, mPAP, and peripheral edema with clinical worsening, with no significant interaction (all interaction *P* > 0.05).

**Table 3:** Predictors of clinical-worsening in patients with FM-PH: univariate analyses and multivariate analyses

Characteristics	Univariate analyses^a^		Multivariate analyses^b^	
	HR(95%CI)	*p*.value	HR(95%CI)	*p*.value
NT-proBNP, pg/ml	1.33 (1.07–1.65)	0.012*		
hs-CRP, mg/L	1.07 (1.01–1.13)	0.014*	1.08 (1.02–1.15)	0.010*
White blood cell, 10^9^/L	1.14 (1.01–1.29)	0.029*		
Monocyte, 10^9^/L	8.47 (1.53–46.90)	0.014*		
SaO_2_, %	0.94 (0.89-1.00)	0.032*		
mPAP, mmHg	1.03 (1.01–1.06)	0.013*	1.03 (1.01–1.06)	0.017*
PVR, wood units	1.07 (1.01–1.14)	0.026*		
RV/LV	4.83 (1.45–16.10)	0.010*		
Peripheral edema	2.14 (1.13–4.07)	0.020*	1.93 (1.01–3.71)	0.040*

*with a P value less than 0.05

CI, confidence interval; FM-PH, fibrosing mediastinitis associated pulmonary hypertension; hs-CRP, high-sensitivity C-reactive protein; HR, hazard ratio; mPAP, mean pulmonary artery pressure; NT-proBNP, N-terminal pro-B-type natriuretic peptide; PVR, Pulmonary vascular resistance; SaO₂, arterial oxygen saturation; RV/LV, right ventricular end-diastolic diameter/left ventricular end-diastolic diameter

a Univariate analyses are based on the complete cases without missing value

b The final variables included in multivariate analyses were chosen by backward-stepwise selection procedure

### Long-term survival

**Fig. 2:**
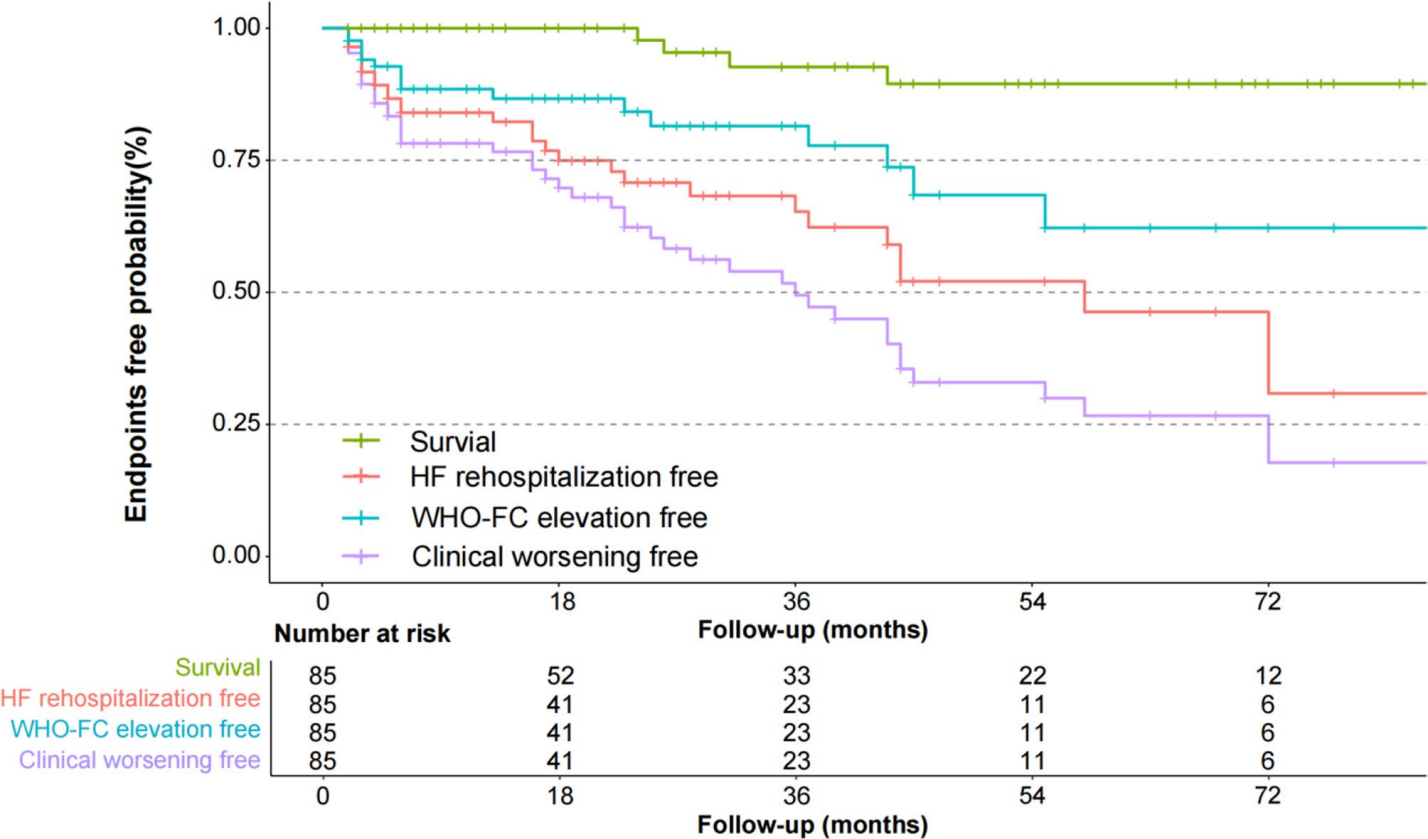
Kaplan-Meier curves for endpoints free rate in patients with FM-PH. FM-PH, fibrosing mediastinitis associated pulmonary hypertension; HF, heart failure; WHO-FC, World Health Organization functional class

**Fig. 3:**
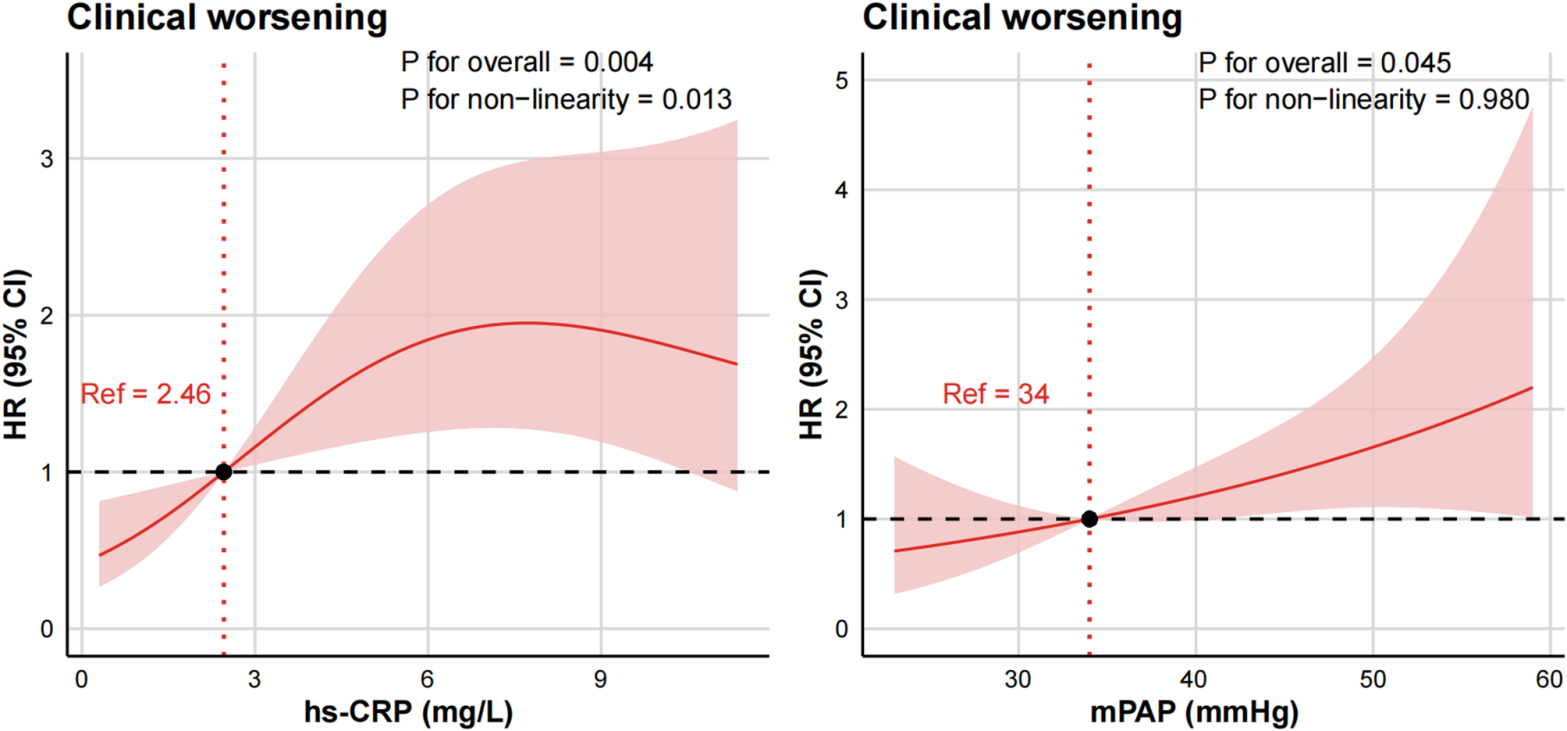
Hazard ratios of clinical worsening as a function of baseline hs-CRP and mPAP. hs-CRP and mPAP as a continuous variable fitted an unadjusted COX regression model using restricted cubic spline regression. hs-CRP, high-sensitivity C-reactive protein; CI, confidence interval; HR, hazard ratio; mPAP, mean pulmonary artery pressure

**Fig. 4:**
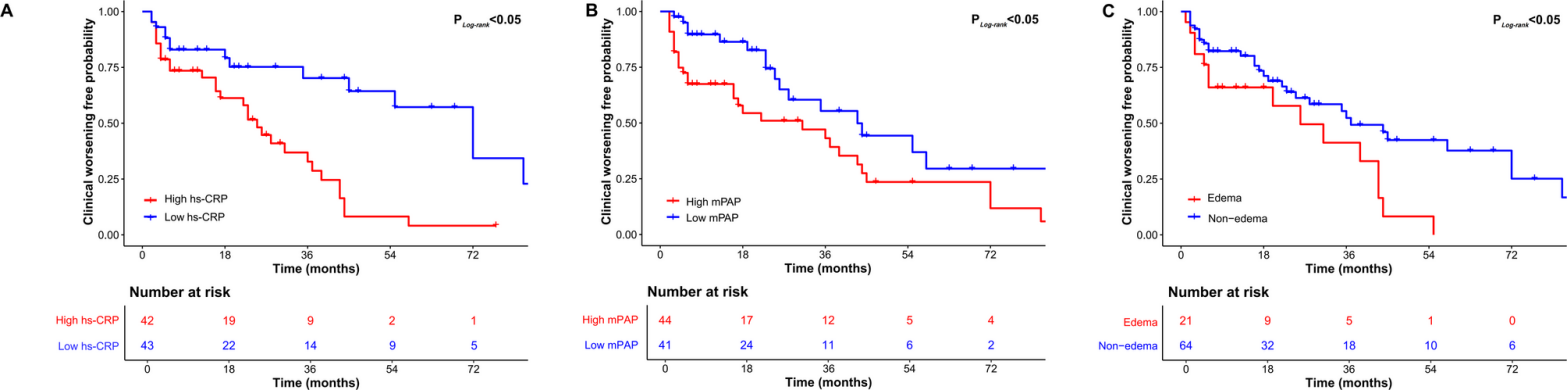
Kaplan-Meier curves for patients with FM-PH classified by baseline levels of hs-CRP, mPAP and peripheral edema. hs-CRP, high-sensitivity C-reactive protein; FM-PH, fibrosing mediastinitis associated pulmonary hypertension; mPAP, mean pulmonary artery pressure

During a median follow-up period of 27 months [IQR 11–55], 48 patients experienced clinical worsening: five died (four from circulatory failure and one from COVID-19), 30 were hospitalized for heart failure, and 18 showed worsening of WHO functional class. Kaplan–Meier analysis revealed 1-, 3-, and 5-year survival rates of 94.5%, 86.3%, and 84.6%, respectively, for patients with FM-PH. The corresponding clinical worsening-free rates at 1, 3, and 5 years were 78.2%, 49.5%, and 26.6% (Fig. 2). hs-CRP and mPAP were next evaluated as continuous variables using a Cox proportional hazards model with restricted cubic spline regression. As shown in Fig. 3, the spline plots demonstrated a significant non-linear association between hs-CRP levels and the HR for clinical worsening (P for overall = 0.004; P for non-linearity = 0.013), while mPAP exhibited a linear relationship (P for overall = 0.045; P for non-linearity = 0.980). Subsequently, patients were stratified into high and low groups based on the identified cut-off values (hs-CRP > 2.46 mg/L vs. ≤ 2.46 mg/L; mPAP > 34 mmHg vs. ≤ 34 mmHg), and Kaplan–Meier survival analyses were conducted. As illustrated in Fig. 4, patients with peripheral edema or increased levels of hs-CRP or mPAP experienced significantly higher rates of clinical worsening compared with those in the lower groups (log-rank *P* < 0.05 for all). Adjusted analyses confirmed that mPAP > 34 mmHg (HR = 2.29, 95% CI 1.05–4.97, *P* = 0.036) and hs-CRP > 2.46 mg/L (HR = 2.24, 95% CI 1.22–4.10, *P* = 0.009) were independent predictors of clinical worsening (Supplementary Table 1).

## Discussion

This retrospective study represents the largest to date focusing on clinical characteristics and long-term outcomes in patients with FM-PH. We assessed risk factors associated with disease progression and poor prognosis and found the following: (1) FM-PH patients who experienced clinical worsening demonstrated more severe systemic inflammation, increased NT-proBNP levels, higher mPAP, and impaired right heart function; (2) Clinical worsening was frequent among FM-PH patients, with elevated hs-CRP, increased mPAP, and the presence of peripheral edema significantly related to worse outcomes, highlighting the value of inflammatory and hemodynamic biomarkers for identifying high-risk individuals; and (3) The 1-, 3-, and 5-year survival rates were 94.5%, 86.3%, and 84.6%, respectively, suggesting generally favorable long-term survival. However, the 5-year clinical worsening-free rate was only 26.6%, underscoring the progressive nature of the disease.

Our study demonstrated that increased hs-CRP levels were strongly associated with clinical deterioration in FM-PH. As a sensitive marker of systemic inflammation, hs-CRP reflects ongoing immune activation [9]. Although the pathogenesis of FM remains incompletely understood, chronic inflammation, immune dysregulation, and fibrosis appear to play central roles. Activated macrophages and T cells release pro-inflammatory cytokines such as TNF-α and IL-6, which stimulate fibroblast proliferation, collagen deposition, and tissue remodeling [10, 11]. For instance, TNF-α can directly activate the TGF-β/Smad signaling pathway, while IL-6 modulates the expression and activity of TGF-β through MAPK/ERK-dependent mechanism [12, 13]. These upstream signals converge on TGF-β, the “master switch” of the fibrotic response. By activating the canonical Smad2/3 pathway, TGF-β not only increases the synthesis of extracellular matrix components (such as collagen and fibronectin) but also reduces extracellular matrix (ECM) degradation by inhibiting the expression of matrix metalloproteinases (MMPs), thereby greatly exacerbating the fibrotic process [14]. These pathological processes contribute to fibrotic changes in the mediastinum, leading to compression of pulmonary vessels and airways, which in turn results in pulmonary hypertension and impaired ventilation.

CRP not only serves as a marker of inflammation but may also actively contribute to the development of vascular lesions [15–17]. It can stimulate endothelial cells to secrete chemokines such as IL-6 and MCP-1, enhance monocyte adhesion and infiltration, and exacerbate vascular inflammation. CRP also promotes the production of reactive oxygen species, drives oxidative stress, and impairs endothelial function [18]. In the pulmonary arteries, CRP activates signaling pathways including ERK1/2, Akt, and NF-κB, which promote the proliferation of pulmonary artery smooth muscle cells (PASMCs) and medial thickening, thereby increasing vascular resistance and contributing to vascular remodeling [19]. Additionally, CRP upregulates the expression of tissue factor (TF), promoting intimal hyperplasia and thrombosis. Through activation of the NF-κB pathway, CRP also contributes to extracellular matrix accumulation and the development of pulmonary fibrosis [20]. Increased CRP levels have been observed in patients with pulmonary hypertension and are positively correlated with right atrial pressure (RAP), WHO-FC, 6MWD, and survival outcomes, suggesting a strong link between inflammation and cardiopulmonary decompensation [21]. Collectively, these findings position hs-CRP as a promising biomarker for disease monitoring and risk stratification in FM-PH.

Moreover, we observed significantly higher leukocyte, neutrophil, and monocyte counts in the clinical worsening group, further highlighting the importance of immune-inflammatory responses in disease progression. Neutrophils, as key components of the innate immune system, can rapidly migrate to sites of inflammation and release neutrophil elastase (NE) and myeloperoxidase (MPO). These enzymes promote PASMC proliferation and migration, activate the Rho kinase pathway, and contribute to vasoconstriction and vascular remodeling [22, 23]. Notably, increased expression of neutrophil-derived MMP-9 has been related to higher mortality in PAH, indicating its role in adverse vascular remodeling and poor outcomes [24, 25]. Monocytes and their differentiated macrophages also play critical roles in the pathogenesis of pulmonary hypertension. They are involved in the early stages of inflammation and later polarize into either M1 or M2 phenotypes, influencing the immune microenvironment and fibrotic pathways [26]. Activated macrophages secrete chemokines such as C-X3-C motif chemokine receptor 1 (CX3CR1) and platelet-derived growth factor (PDGF), which promote PASMC proliferation and migration [27]. Additionally, chemokine receptors such as C-C chemokine receptor type 2 (CCR2) and C-C chemokine receptor type 5 (CCR5) facilitate interactions between macrophages and PASMCs, contributing to pulmonary vascular remodeling and increased pulmonary arterial pressure [28].

Hemodynamic evaluation offered additional prognostic insights. Patients who experienced clinical worsening had significantly increased mPAP and PVR, confirming that increased pulmonary vascular burden is directly associated with adverse clinical outcomes. Beyond objective measurements, peripheral edema—an indicator of right heart dysfunction—was also identified as an independent risk factor for clinical worsening. These findings highlight the importance of continuous monitoring of both hemodynamic parameters and clinical signs in the management of FM-PH.

Currently, data on long-term outcomes in FM-PH remains limited. In our cohort, survival rates were notably higher than those reported in a prospective French study of 27 FM-PH patients, which documented 1-, 3-, and 5-year survival rates of 88%, 73%, and 56%, respectively. This discrepancy may be attributed to differences in patient management, as individuals in our study were more likely to have received pulmonary artery interventions and had relatively lower baseline mPAP, both of which may have contributed to better outcomes [7]. Furthermore, a retrospective study from the Mayo Clinic involving 80 FM patients reported only five deaths over a 10-year follow-up period, with just two deaths directly related to FM. These findings suggest that FM, overall, may follow a more indolent course than previously believed. However, the referenced study did not specifically identify patients with concurrent pulmonary hypertension, likely underestimating the risk in this high-risk subgroup [29]. Despite the encouraging survival observed in our cohort, the low 5-year clinical worsening-free rate underscores the persistent risk of disease progression. This finding reinforces the importance of ongoing monitoring and the implementation of personalized treatment strategies to slow disease deterioration and optimize long-term outcomes.

This study has several limitations. First, FM-PH is an extremely rare and understudied subtype of pulmonary hypertension. Although this represents the largest cohort reported to date, the overall sample size remains relatively small, and the number of death events was limited. This restricted our ability to conduct a robust analysis of mortality—a definitive clinical endpoint—using the Cox proportional hazards model. Consequently, we focused on clinical worsening as the primary outcome and were unable to further adjust for or validate prognostic factors specifically related to mortality in FM-PH. Second, the diagnosis of FM was mainly based on CT imaging features rather than histopathological confirmation. Invasive procedures such as mediastinoscopy or endobronchial ultrasound-guided transbronchial needle aspiration were not routinely performed owing to the high risk associated with these techniques in patients with coexisting pulmonary hypertension. Third, levels of hs-CRP and mPAP can fluctuate throughout the disease course. However, this study relied solely on baseline measurements and did not evaluate longitudinal changes during follow-up. As such, the long-term prognostic value of these biomarkers requires further validation in large-scale prospective studies.

## Conclusion

We conducted the largest multicenter retrospective study to date on the clinical characteristics, risk factors, and long-term outcomes of FM-PH in China. Elevated hs-CRP, increased mPAP, and the presence of peripheral edema were independently associated with clinical worsening. Although the 1-, 3-, and 5-year overall survival rates were relatively favorable, the 5-year clinical worsening-free survival rate remained unsatisfactory. Further prospective studies with larger cohorts are warranted to validate and expand upon these findings.

## Supplementary Information

Below is the link to the electronic supplementary material.

Supplementary Material 1

## Data Availability

The datasets used and/or analyzed during the current study are available from the corresponding author on reasonable request.

## Abbreviations

BHLN: Bilateral hilar lymph nodes
BMI: Body mass index
BPA: Balloon pulmonary angioplasty
CTPA: Computed tomography pulmonary angiography
CRP: C-reactive protein
DLCO: Diffusion capacity of carbon monoxide
D_MPA_: Diameter of main pulmonary artery
ESR: Erythrocyte sedimentation rate
FM: Fibrosing mediastinitis
FM-PH: Fibrosing mediastinitis associated pulmonary hypertension
FVC: Forced vital capacity
FEV1: Forced expiratory value in 1 s
hs-CRP: High-sensitivity C-reactive protein
LLL: Left lower lobe
LPA: Left pulmonary artery
LUL: Left upper lobe
LVED: Left ventricular end-diastolic diameter
LVEF: Left ventricular ejection fraction
MLN: Mediastinal lymph nodes
MPA: Main pulmonary artery
mPAP: Mean pulmonary artery pressure
mRAP: Mean right atrial pressure
NT-proBNP: N-terminal pro-brain natriuretic peptide
PAC: Pulmonary arterial compliance
PaCO_2_: Partial pressure of carbon dioxide
PaO_2_: Partial pressure of oxygen
PAWP: Pulmonary artery wedge pressure
PH: Pulmonary hypertension
PV: Pulmonary vein
PVR: Pulmonary vascular resistance
RLL: Right lower lobe
RML: Right middle lobe
RPA: Right pulmonary artery
RUL: Right upper lobe
RVED: Right ventricular end-diastolic diameter
RV/LV: Right ventricular end-diastolic diameter/left ventricular end-diastolic diameter
SaO_2_: Arterial oxygen saturation
sPAP: Systolic pulmonary arterial pressure
SpaO_2_: Pulmonary arterial oxygen saturation
SvO_2_: Mixed venous oxygen saturation
SVC: Superior vena cava
TC: Total cholesterol
TLC: Total lung capacity
V_TR_: Velocity of tricuspid regurgitation
% pred: Percent of predicted value
WHO-FC: World Health Organization functional class

## References

[1] Garrana SH, Buckley JR, Rosado-de-Christenson ML, Martínez-Jiménez S, Muñoz P, Borsa JJ. Multimodality imaging of focal and diffuse fibrosing mediastinitis. Radiographics. 2019;39(3):651–67. 30951437 10.1148/rg.2019180143

[2] Dong S, Dai X, Jiang Y, Zheng J. Idiopathic fibrous mediastinitis with refractory pleural effusion: a case report and literature review. J Int Med Res. 2021;49(8):3000605211040264. 34459275 10.1177/03000605211040264 PMC8408902

[3] Wang A, Su H, Duan Y, Jiang K, Li Y, Deng M, Long X, Wang H, Zhang M, Zhang Y, et al. Pulmonary hypertension caused by fibrosing mediastinitis. JACC Asia. 2022;2(3):218–34. 36338410 10.1016/j.jacasi.2021.11.016 PMC9627819

[4] Manyeruke FD, Perumal R, Symons G, Mottay L. Idiopathic fibrosing mediastinitis. Afr J Thorac Crit Care Med. 2021;27(2). 10.7196/AJTCCM.2021.v27i2.064 PMC8327681 34430869

[5] Liu T, Gao L, Xie S, Sun H, Liu M, Zhai Z. Clinical and imaging spectrum of tuberculosis-associated fibrosing mediastinitis. Clin Respir J. 2018;12(5):1974–80. 29356415 10.1111/crj.12766

[6] Humbert M, Kovacs G, Hoeper MM, Badagliacca R, Berger RMF, Brida M, Carlsen J, Coats AJS, Escribano-Subias P, Ferrari P, et al. 2022 ESC/ERS guidelines for the diagnosis and treatment of pulmonary hypertension. Eur Heart J. 2022;43(38):3618–731. 36017548 10.1093/eurheartj/ehac237

[7] Seferian A, Steriade A, Jaïs X, Planché O, Savale L, Parent F, Amar D, Jovan R, Fadel E, Sitbon O, et al. Pulmonary hypertension complicating fibrosing mediastinitis. Med (Baltim). 2015;94(44):e1800. 10.1097/MD.0000000000001800 PMC4915879 26554778

[8] Mukherjee M, Rudski LG, Addetia K, Afilalo J, D’Alto M, Freed BH, Friend LB, Gargani L, Grapsa J, Hassoun PM, et al. Guidelines for the echocardiographic assessment of the right heart in adults and special considerations in pulmonary hypertension: recommendations from the American society of echocardiography. J Am Soc Echocardiogr. 2025;38(3):141–86. 40044341 10.1016/j.echo.2025.01.006

[9] Pepys MB, Hirschfield GM. C-reactive protein: a critical update. J Clin Invest. 2003;111(12):1805–12. 12813013 10.1172/JCI18921 PMC161431

[10] Steele H, Cheng J, Willicut A, Dell G, Breckenridge J, Culberson E, Ghastine A, Tardif V, Herro R. TNF superfamily control of tissue remodeling and fibrosis. Front Immunol. 2023;14:1219907. 37465675 10.3389/fimmu.2023.1219907 PMC10351606

[11] Li Y, Zhao J, Yin Y, Li K, Zhang C, Zheng Y. The role of IL-6 in fibrotic diseases: molecular and cellular mechanisms. Int J Biol Sci. 2022;18(14):5405–14. 36147459 10.7150/ijbs.75876 PMC9461670

[12] Sullivan DE, Ferris M, Pociask D, Brody AR. Tumor necrosis factor-alpha induces transforming growth factor-beta1 expression in lung fibroblasts through the extracellular signal-regulated kinase pathway. Am J Respir Cell Mol Biol. 2005;32(4):342–9. 15653932 10.1165/rcmb.2004-0288OC

[13] Luckett-Chastain LR, Gallucci RM. Interleukin (IL)-6 modulates transforming growth factor-beta expression in skin and dermal fibroblasts from IL-6-deficient mice. Br J Dermatol. 2009;161(2):237–48. 19438433 10.1111/j.1365-2133.2009.09215.x PMC2766075

[14] Henderson NC, Rieder F, Wynn TA. Fibrosis: from mechanisms to medicines. Nature. 2020;587(7835):555–66. 33239795 10.1038/s41586-020-2938-9 PMC8034822

[15] Dolenc J, Šebeštjen M, Vrtovec B, Koželj M, Haddad F. Pulmonary hypertension in patients with advanced heart failure is associated with increased levels of interleukin-6. Biomarkers. 2014;19(5):385–90. 24831174 10.3109/1354750X.2014.918654

[16] Wynants M, Quarck R, Ronisz A, Alfaro-Moreno E, Van Raemdonck D, Meyns B, Delcroix M. Effects of C-reactive protein on human pulmonary vascular cells in chronic thromboembolic pulmonary hypertension. Eur Respir J. 2012;40(4):886–94. 22267767 10.1183/09031936.00197511

[17] Santos-Gomes J, Gandra I, Adão R, Perros F, Brás-Silva C. An overview of Circulating pulmonary arterial hypertension biomarkers. Front Cardiovasc Med. 2022;9:924873. 35911521 10.3389/fcvm.2022.924873 PMC9333554

[18] Li J, Li JJ, He JG, Nan JL, Guo YL, Xiong CM. Atorvastatin decreases C-reactive protein-induced inflammatory response in pulmonary artery smooth muscle cells by inhibiting nuclear factor-kappaB pathway. Cardiovasc Ther. 2010;28(1):8–14. 20074254 10.1111/j.1755-5922.2009.00103.x

[19] Li J, Luo SH, Tang Y, Li JJ. C-reactive protein induces pulmonary artery smooth cell proliferation via modulation of ERK1/2, Akt and NF-kappaB pathways. Clin Lab. 2014;60(8):1357–63. 25185422 10.7754/clin.lab.2013.130111

[20] Cirillo P, Golino P, Calabrò P, Calì G, Ragni M, De Rosa S, Cimmino G, Pacileo M, De Palma R, Forte L, et al. C-reactive protein induces tissue factor expression and promotes smooth muscle and endothelial cell proliferation. Cardiovasc Res. 2005;68(1):47–55. 16023093 10.1016/j.cardiores.2005.05.010

[21] Quarck R, Nawrot T, Meyns B, Delcroix M. C-reactive protein: a new predictor of adverse outcome in pulmonary arterial hypertension. J Am Coll Cardiol. 2009;53(14):1211–8. 19341863 10.1016/j.jacc.2008.12.038

[22] Kim YM, Haghighat L, Spiekerkoetter E, Sawada H, Alvira CM, Wang L, Acharya S, Rodriguez-Colon G, Orton A, Zhao M, et al. Neutrophil elastase is produced by pulmonary artery smooth muscle cells and is linked to neointimal lesions. Am J Pathol. 2011;179(3):1560–72. 21763677 10.1016/j.ajpath.2011.05.051 PMC3157285

[23] Klinke A, Berghausen E, Friedrichs K, Molz S, Lau D, Remane L, Berlin M, Kaltwasser C, Adam M, Mehrkens D, et al. Myeloperoxidase aggravates pulmonary arterial hypertension by activation of vascular Rho-kinase. JCI Insight. 2018;3(11). 10.1172/jci.insight.97530 PMC6124430 29875311

[24] Jonigk D, Golpon H, Bockmeyer CL, Maegel L, Hoeper MM, Gottlieb J, Nickel N, Hussein K, Maus U, Lehmann U, et al. Plexiform lesions in pulmonary arterial hypertension composition, architecture, and microenvironment. Am J Pathol. 2011;179(1):167–79. 21703400 10.1016/j.ajpath.2011.03.040 PMC3123793

[25] Zhang R, Zhang J, Zhang YL, Gong SG, Zhao QH, Wang XJ, Zhao JY, Jiang R, Qiu HL, Li HT, et al. Single-Cell transcriptome analysis of peripheral neutrophils from patients with idiopathic pulmonary arterial hypertension. Hypertension. 2023;80(8):1784–94. 37313754 10.1161/HYPERTENSIONAHA.123.21142

[26] Trial J, Cieslik KA, Haudek SB, Duerrschmid C, Entman ML. Th1/M1 conversion to th2/m2 responses in models of inflammation lacking cell death stimulates maturation of monocyte precursors to fibroblasts. Front Immunol. 2013;4:287. 24065967 10.3389/fimmu.2013.00287 PMC3776235

[27] Yaku A, Inagaki T, Asano R, Okazawa M, Mori H, Sato A, Hia F, Masaki T, Manabe Y, Ishibashi T, et al. Regnase-1 prevents pulmonary arterial hypertension through mRNA degradation of Interleukin-6 and Platelet-Derived growth factor in alveolar macrophages. Circulation. 2022;146(13):1006–22. 35997026 10.1161/CIRCULATIONAHA.122.059435

[28] Abid S, Marcos E, Parpaleix A, Amsellem V, Breau M, Houssaini A, Vienney N, Lefevre M, Derumeaux G, Evans S, et al. CCR2/CCR5-mediated macrophage-smooth muscle cell crosstalk in pulmonary hypertension. Eur Respir J. 2019;54(4). 10.1183/13993003.02308-2018 31320454

[29] Peikert T, Colby TV, Midthun DE, Pairolero PC, Edell ES, Schroeder DR, Specks U. Fibrosing mediastinitis: clinical presentation, therapeutic outcomes, and adaptive immune response. Med (Baltim). 2011;90(6):412–23. 10.1097/MD.0b013e318237c8e6 22033450

