# Supplementary material for: Long-term outcomes and prognostic predictors in patients with fibrosing mediastinitis associated pulmonary hypertension: a multicenter cohort study
Source: Orphanet J Rare Dis. 2025 Nov 12;20:576. doi: 10.1186/s13023-025-04066-8 (PMC12613404; doi:10.1186/s13023-025-04066-8)
Supplement: Supplementary file 1 — Supplementary Material 1 [file 13023_2025_4066_MOESM1_ESM.docx]

**Supplementary Figure 1** Subgroup and interaction analyses of the association between hs-CRP, mPAP and peripheral edema and clinical worsening.


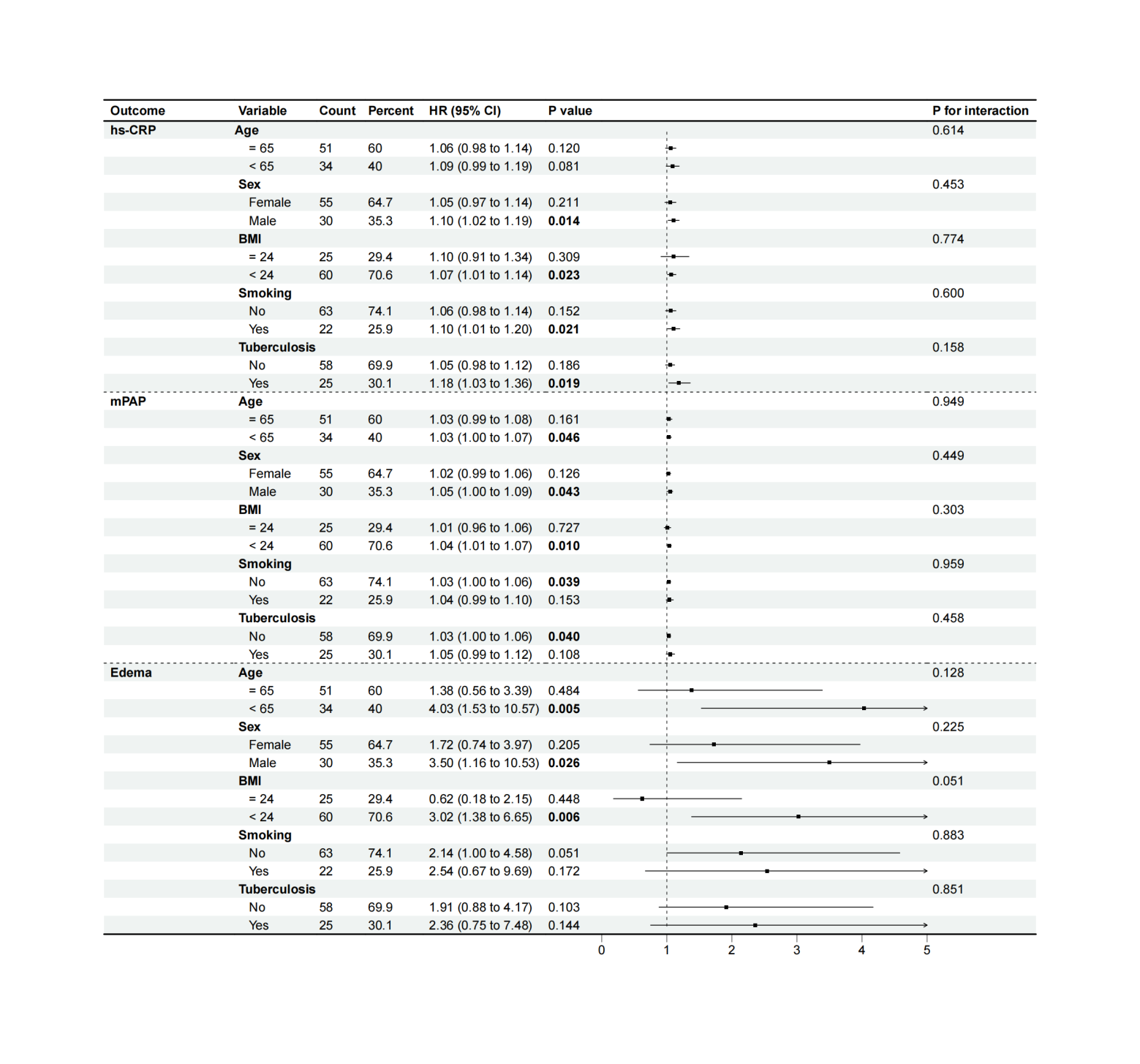
BMI, body mass index; CI, confidence interval; CRP, C-reactive protein; HR, hazard ratio; mPAP, mean pulmonary artery pressure

**Supplementary table 1** Multivariate analyses assessing the association between categorical hs-CRP and mPAP and clinical worsening in patients with FM-PH.

| Characteristics | Univariate analyses^a^ | | Multivariate analyses^b^ | |
| --- | --- | --- | --- | --- |
|  | HR(95%CI) | *p.value* | HR(95%CI) | *p.value* |
| NT-proBNP, pg/ml | 1.33 (1.07-1.65) | 0.012* |  |  |
| hs-CRP, high hs-CRP vs. low hs-CRP mg/L^c^ | 3.23 (1.67-6.23) | ＜0.001* | 2.29 (1.05-4.97) | 0.036* |
| White blood cell, 10^9^/L | 1.14 (1.01-1.29) | 0.029* |  |  |
| Monocyte, 10^9^/L | 8.47 (1.53-46.90) | 0.014* |  |  |
| SaO_2_, % | 0.94 (0.89-1.00) | 0.032* |  |  |
| mPAP, high mPAP vs. low mPAP, mmHg | 2.14 (1.18-3.88) | 0.013* | 2.24 (1.22-4.10) | 0.009* |
| PVR, wood units | 1.07 (1.01-1.14) | 0.026* |  |  |
| RV/LV | 4.83 (1.45-16.10) | 0.010* |  |  |
| Peripheral edema | 2.14 (1.13-4.07) | 0.020* | 2.11 (1.02-4.36) | 0.043* |

*with a P value less than 0.05

CI, confidence interval; FM-PH, fibrosing mediastinitis associated pulmonary hypertension; hs-CRP, high-sensitivity C-reactive protein; HR, hazard ratio; mPAP, mean pulmonary artery pressure; NT-proBNP, N-terminal pro-B-type natriuretic peptide; PVR, Pulmonary vascular resistance; SaO₂, arterial oxygen saturation; RV/LV, right ventricular end-diastolic diameter/left ventricular end-diastolic diameter.

a Univariate analyses are based on the complete cases without missing value.

b The final variables included in multivariate analyses were chosen by backward-stepwise selection procedure.

c High hs-CRP group was defined as patients with hs-CRP > 2.46 mg/L, low hs-CRP was defined as patients with hs-CRP ≤ 2.46 mg/L; High mPAP group was defined as patients with mPAP > 34 mmHg, low mPAP was defined as patients with mPAP ≤ 34 mmHg
